# Navigating the feminine in massively multiplayer online games: gender in *World of Warcraft*

**DOI:** 10.3389/fpsyg.2013.00903

**Published:** 2013-12-04

**Authors:** Audrey L. Brehm

**Affiliations:** Department of Sociology, University of Colorado at Colorado SpringsColorado Springs, CO, USA

**Keywords:** *World of Warcraft*, MMORPG, sexism, online video games, gender, cyberbullying, disinhibition

## Abstract

The objective of the study is to present and discuss attitudes, perceptions and opinions about sexism and gendered play in the massively multiplayer online roleplaying game (MMO), *World of Warcraft*. Through the use of an online survey which includes both multiple choice questions and open-ended questions, the research discusses the major themes and findings expressed by the *World of Warcraft* forum users (*N* = 294). The descriptive statistical findings presented are derived from the multiple choice questions. Within the sample, the results reveal that sexism is a contentious topic in the *World of Warcraft* community. 63.6% (*n* = 75) of female respondents reported experiencing sexism within the game. 27.5% (*n* = 44) of male respondents and 45.3% (*n* = 53) of female respondents believe that sexism is a problem in the game. Overall, 64.4% (*n* = 183) of the respondents reported sexism as a non-issue in the game. Themes surrounding the topic of sexism experienced within the game are presented based on frequency of homogenous responses. Based on the multiple choice questions and the open-ended questions, the research argues that sexism and gendered play in gaming should be studied more closely, as the results reveal that many MMO players are affected negatively by it.

## Introduction

Gender inequality in contemporary society is continually an important topic of study in multiple disciplines. The study of gender in relation to computer games merits more growth as it is an underdeveloped topic. An increasingly popular field of study examines the dynamics of female participation in online gaming. Arguably, societal issues, cultural norms and gender roles translate themselves into the social online gaming world (Malpas, 2009). Video and computer games can act as a cultural learning tool, especially for young people. Just as media representations can influence conceptions of culturally sanctioned gender roles, video games may have this same effect. The theory attributed to this way of learning is referred to as social learning theory (Bandura, 1965; Durkin, 1985; Beasley and Standley, 2002). In social learning theory, children learn to develop certain gendered behaviors and beliefs according to what they are exposed to on a daily basis such as TV, video games, etc. Relatedly, older and more recent research indicates a strong correlation between consumption of media and attitudes of acceptable gendered behavior and clothing (Beasley and Standley, 2002). Based on this knowledge, the review of the literature works from the perspective that media and online gaming can create and reinforce gendered behaviors.

The literature review seeks to explore how femininity is received in online games and more specifically, massively multiplayer online games or MMOs. This includes gender stereotyping, how female gamers are treated, how female characters in games are displayed and created, what can be done to make the online gaming experience more inclusive and how the topic of gender harassment might be related to cyberbullying.

There is often an assumption that the majority of gamers are male, yet this is misleading information. Recent statistics have revealed that about 45% of all gamers are female (ESA, 2013). Additionally, a study performed in 2010 by the Entertainment Software Association (2010) found that 42% of online game players are female. Currently, *World of Warcraft*, the game of focus for this study, has a total of 7.7 million subscribers—a number not to be scoffed at ^1^^,^^2^. Past research by Yee (2008) found that about 15 percent of the MMO player base is female. This number is not exact; the total number of female MMO players is unknown at this point in time. While the number is modest, it still indicates a significant amount of female players. Based on these statistics, it is prudent that more research should look into how femininity is received in computer games.

### The relationship of harassment and cyberbullying in MMOs

There is sparse research that looks at the relation of cyberbullying to the online gaming world. There is, however, research both supporting and refuting violent video games as a predictor of aggressive tendencies in players (whether *World of Warcraft* is a violent video game is debatable) (Dietz, 1998; Anderson, 2004; Dill et al., 2008; Markey and Markey, 2010). Markey and Markey (2010) discuss reasons why it is misguided to attribute aggressive behavior to violent video games. They point out that, “although many youths who have engaged in violent school rampages were video game players, most also possessed maladaptive personality traits and characteristics” (p. 82). Using this knowledge, perhaps some players in online video games, such as MMOs, are perpetrators of cyberbullying/sexism/harassment not because of the gaming environment itself but because of preexisting personality traits and beliefs which are often influenced by societal norms (masculinity, power, male dominance, etc.). In one of the few works to examine cyberbullying in a gaming environment, Coyne et al. (2009) discuss how cyberbullying translates into gaming terms. Instead of cyberbullying, we might use the terms griefing or flaming. While griefing is a form of harassment, it is defined as making use of the game structure or physics in order to cause distress (Warner and Raiter, 2005; Coyne et al., 2009). Whether this could be a form of sexism in online gaming is something to be explored. More persistently, the problem of cyberbullying in online games occurs in the form of flaming. Flaming is defined as, “an uninhibited expression of hostility, such as swearing, calling names, ridiculing and hurling insults toward another person, his/her character, religion, race, intelligence and physical or mental ability” (Kayany, 1998, p. 1138). Of course in this context, I add gender to the list. It can be argued that flaming and griefing are a form of cyberbullying, but this is certainly up for future research to explore. Research suggests, however, that online gaming is not immune to sexist attitudes and behaviors and whether these are forms of cyberbullying remains unseen.

### The prevalence of sexism

A common way to assert masculinity in our culture is to demonize and portray anything that does not fit into the masculine model as something to be avoided, such as femininity. Hypermasculinity allows for an abundance of sexism in online gaming. It has been well-documented that sexism is extremely prevalent (King et al., 1991; Herring, 1999; Bertozzi, 2008; Dill et al., 2008; Jenkins and Cassell, 2008; Gray, 2012; Salter and Blodgett, 2012; O'Leary, 2012; Braithwaite, 2013). One can look to several incidents of public witch hunts of women who have spoken out against sexism in the gaming world. Salter and Blodgett (2012) discuss an incident whereby a prominent gaming website came under attack for making rape jokes. As the authors state, those who spoke out against the rape jokes were “belittled, verbally assaulted, and harassed from many areas within the hardcore gaming public” Salter and Blodgett (2012, p. 411). In another recent sexist outburst of harassment, Anita Sarkeesian, a media critic and feminist activist was assailed by the gaming public for her choice to create well-researched videos exploring tropes and stereotypes of female characters in video games. The attacks ranged from harassment and threats to cartoon depictions of her imagined rape (Graham, 2012). These examples are not relegated as isolated incidents, for example, female gamers report and post occasions of overt sexism on a website aptly named “Fat, Ugly, or Slutty”^3^. These posts include screenshots and audio recordings of sexist attacks in games (Salter and Blodgett, 2012). It is no wonder then that female gamers have expressed reluctance to engage in gendered discourse and avoid “outing” themselves as female (Dill et al., 2008; Salter and Blodgett, 2012).

A recent study examined the responses of male gamers to both female and male voices over voice chat in the game *Halo 3.* The objective was to play in games with other players and broadcast generic statements over voice chat such as “good game” or “hi everybody.” The researchers found that the female voice received three times the amount of directed negative comments than the male voice. The researchers were also careful to account for skill. The researcher behind the actual playing portion of the research had an acceptable skill level and played as both the male and female voice. As stated, “With regard to the connection between skill level and comments, this study did not find an overall correlation between skill level and negative comments in the three voice conditions” (Kuznekoff and Rose, 2012). The study is important because it provides concrete evidence of how female players are received in this particular online game.

In the offline world, women are often given sexist roles in real life gaming events (e.g., gaming conventions and competitive gaming). These tend to be very specific roles. As Taylor et al. (2009) state, these roles are “tenuously maintained within a community that most commonly reads female participation in sexualized terms: female players risk being labeled as “*Halo* hoes,” mothers at events describe themselves as “cheerleaders,” and promotional models become “booth babes”—all supportive, subordinate roles” (p. 243). These roles work to support the dismissal of women as gamers. A popular phrase in online gaming is, “There are no women on the internet” (Herring, 1999; Taylor, 2006; Salter and Blodgett, 2012). As evidenced by these examples, online video and computer games allow dominant, masculine ideologies to be reproduced, and this dominant ideology is often a sexist one (Gray, 2012).

### Gender stereotyping in online games

With an ever-growing base of female players, it is imperative to consider the implications of gendered cultural presumptions. As we know from previous research, stereotypes from video games may reinforce negative gender stereotypes, schemas, and scripts in social relations between men and women. Ambivalent Sexism Theory explicates the idea of gender norms and the resulting negative implications for society. It describes society as valuing male traits such as aggression and dominance and therefore places men in a superior position than women. Women, in contrast, are valued for being submissive and likable and are often found in subordinate roles (Dill et al., 2008). In a similar vein of thinking, Gender Schema Theory exposes stereotypes and sexual interactions in media that categorizes women as submissive, sexual, and less intelligent than men (Downs and Smith, 2009). We often see female gamers receiving help from male gamers, whether wanted or not. The stereotype that women are subordinate, less experienced, and less intelligent may indicate why men might feel as though they need to fill a “white knight” role. As stated by Eklund (2011), “To see female gamers as in need of help and guidance is a strategy to keep control over the game; to view women as less competent is to make them harmless” (p. 333). Applying both Ambivalent Sexism Theory and Gender Schema Theory to computer and video games immediately produces countless examples of gender stereotyping; molds that are difficult to break away from in the gaming industry.

Hypermasculinity stands as one of these molds that male gamers might find hard to break away from. Hypermasculinity, in this context, is an amplification of “masculine” cultural stereotypes (Parrott and Zeichner, 2008). It is often associated with “women-hostile concepts of masculinity” and relies on masculine physical traits and behaviors which can result in dismissal or hostility toward expressions of femininity (Salter and Blodgett, 2012, p. 402). One can simply look at the preponderance of rape references among gamers for an illustration of hostile behaviors (Salter and Blodgett, 2012). Hypermasculinity provides a ripe environment for misogynistic practices and behaviors. It is postulated that violations of male gender roles (such as femininity) produces a significant threat to the self-concept of men, especially to those who adhere to extreme male gender roles (Parrott and Zeichner, 2008). Many researchers discuss the idea of a related concept, hegemonic masculinity, as an indicator of male dominance in gaming culture. Connell (1995) describes hegemonic masculinity as “the configuration of gender practice which embodies the currently accepted answer to the problem of the legitimacy of patriarchy, which guarantees (or is taken to guarantee) the dominant position of men and the subordination of women” (p. 77). Hegemonic masculinity is never a static product, it reproduces itself through interaction; it is constructed in social situations (Kendall, 2000). Because MMOs are male dominated and generally social games, hegemonic masculinity and hypermasculinity have the ability to permeate every aspect of these games. The fear of retaliation from male counterparts in online games for not adhering to the male norm can result in negative outcomes such as not being able to reveal a female gender identity. More specifically, female players often feel as though expectations of them will decrease or fellow gamers will cease playing with them if their identity is revealed (Yee, 2008; Eklund, 2011). For example, it has been shown that women who participated in online discussion forums on equal footing as men were consistently ignored, trivialized, vilified, etc. (Herring, 1999).

In contrast to expected norms of masculinity, when female characters are included in games, they tend to fulfill specific “feminine” roles. Alongside a general lack of female characters in computer and video games, these characters find themselves in stereotypical “feminine” roles such as the damsel in distress or the “prize” for completing game objectives (Bryce and Rutter, 2003). Frequently female characters are found in background roles, whereby the woman is simply a supporting character in relation to a male character. Other common roles they fill include performing as objects for pleasure or antagonistic enemies (Salter and Blodgett, 2012). Female characters are rarely dynamic and complex and are still a minority as main characters. The problem continues to be that issues of gender are put up against the norm of a white, heterosexual, male model (Eklund, 2011). With male ownership over production and consumption, it is no surprise that gender stereotyping continues to exist in online gaming (Taylor et al., 2009).

### Sexualized female characters

When examining specifically the way female characters are presented in online gaming, a few trends emerge. Female characters are often displayed in clothing or armor that is considered to be seductive and objectifying (Beasley and Standley, 2002; Kennedy, 2002). Differences between gendered avatars are often emphasized rather than diminished. A study performed in 2007 revealed that within a total of 479 images analyzed in popular gaming magazines, 59.9% of the female characters were found to be sexualized, whereas less than 1% of the male characters were rated as being sexualized (Dill and Thill, 2007). Other studies present similar results and even a correlation between exposure to sex-stereotyped video game characters and tolerance of sexual harassment (Beasley and Standley, 2002; Dill et al., 2008; Downs and Smith, 2009). Female gamers are left with character options that are far from realistic and more often than not, sexualized (Salter and Blodgett, 2012).

Often, female avatars will sport enormous breasts while male avatars are shown as muscular and heroic (Bertozzi, 2008). Interestingly, male avatars are not immune to unrealistic body portrayals. Ideals of physical appearance affect both male and female avatars. With that being said, male avatars may be presented as muscular and heroic, adhering to media ideals, but are rarely portrayed as objects of sexual desire (Taylor, 2003; Dill and Thill, 2007). This does not mean that men are not affected negatively; there exists research that connects ideals of physical appearance in the media to poor body image (Tiggemann and McGill, 2004; Dohnt and Tiggemann, 2006). Both males and females might feel as though they are not living up to culturally prescribed standards. Gerbner's Cultivation Theory is applicable here with the idea that, “mass media creates a worldview more consistent with media's distortion of reality than with reality itself” (Dill and Thill, 2007, p. 854).

### What deters women from playing MMOs?

What is it that makes the MMO female player base so small? One explanation might be that women remain marginalized in “hardcore” gaming communities. The myth perpetrated by online gaming communities is that there are no women on the internet which helps to keep female gamers and femininity invisible. There are gamers that challenge these perceptions, however, engaging in discourse around gender and sexism generally results in belittlement and harassment (Salter and Blodgett, 2012). Female characters are also marginalized as male characters are much more likely to appear and hold prominent roles in games (Beasley and Standley, 2002; Bryce and Rutter, 2003; Downs and Smith, 2009; Eklund, 2011). In fact, a study examining sexist depictions of video game characters revealed that male characters are given greater respect than female characters (Dill and Thill, 2007). It is suggested that because games tend to feature vastly more male characters, young males are attracted to play these games and grow up to be more likely to become part of the game making process and therefore continue the cycle of male dominance in the gaming industry (Williams et al., 2009b).

Unwillingness on the part of both male and female gamers to cross traditional gendered lines of play deters women from playing games that are labeled as a masculine pastime (Royse et al., 2007; Bertozzi, 2008; Williams et al., 2009a; Eklund, 2011). Challenging these gendered lines and playing on the perceived turf of men and consequently being “punished” for doing so can be a factor in deterring women from gaming (Bertozzi, 2008). Interestingly, it is not generally the mechanics of the game that deter women from playing; it is the game culture (Yee, 2008). Recurring issues in online gaming culture include sexual harassment, sexism, and sexist imagery (Bryce and Rutter, 2003; Dill et al., 2008; Williams et al., 2009a; Gray, 2012). The use of femininity as an aesthetic rather than an agentic continues to exclude women as legitimate players in games (Kennedy, 2002). The contention is not that females do not play computer games, because they do; females are a strong player base for casual games and a growing proportion of MMO players. The problem is that more complex games, like MMOs are just simply not marketed toward women and cultural expectations of gendered play continue to label “hardcore” games as a masculine pursuit.

### Where the research is needed

The results of previous studies indicate an apparent point at issue pertaining to gendered interaction in both computer and video games. The research on the sexualized representations of female characters is becoming well-fleshed out and has great potential to influence how games of the future are created. However, what are lacking are documented opinions and experiences of gamers with regards to gendered interactions. More specifically, common themes and theories surrounding what sexism looks like in online games should be researched so that game developers may take steps to improve their games for all genders. MMOs, because they have a large pool of players that interact on a daily basis, provide a beneficial opportunity for both researchers and respondents to examine the dynamics of gendered play. Using a partial grounded theory and narrative analysis approach, the aim of this research is to explore the dynamics of gender and sexism from both male and female gamer perspectives, within the game, *World of Warcraft.* In this research, sexism will be generally defined as “the differential valuing of one sex, in this case, men, over the other” (Renzetti and Curran, 2003, p. 9). The theme of sexism is specifically used because it has been documented as an issue and to use different language, in the opinion of the researcher, would be straying away from an apparent problem in the gaming community. In addition, both previous research and participant responses indicate that sexism is experienced primarily by female gamers; the research focuses on female *experiences and perspectives* of sexism, but includes male *perspectives* as well. Therefore, the research questions are as follows:

RQ1: How is the female gender treated in *World of Warcraft*?
RQ2: What are the basic differences in opinion of sexism between male and female respondents? Do male and female players view the issue of sexism differently?
RQ3: What do typical female experiences of sexism look like in *World of Warcraft*?
RQ4: What do respondents think can be done about sexism in *World of Warcraft*? Do they view it as an issue?

## Materials and methods

### Respondents

Participants were asked to respond to questions regarding sexism in the game, *World of Warcraft*. A survey link with a consent form and instructions was posted on the *World of Warcraft* forum. The website SurveyMonkey was chosen as the survey site. Participants were self-selected in that the researcher did not choose who would respond to the survey. Those who were under the age of 18 were not included in the survey. The consent form asked for respondents to confirm that they were 18 and older. Respondents, except for the initial question of consent, had the option to skip all questions. The survey saw a total of *N* = 294 respondents with 166 (58.2%) as male, 119 (41.8%) as female, and 7 (2.4%) reporting as not identifying as either male or female. According to surveys performed in the past by Yee (2008), the percentage of female *World of Warcraft* players is around 15%. The significant increase in the percentage of female players in this survey might be explained in a couple ways. First, this is a relatively small sample; the results may be less reliable in generalizing to the entire population. The surveys performed by Yee received ~2000–4000 respondents in each survey phase, making it much more reliable in generalizing to the population (Yee, 2008). Second, the forum post specifically identified sexism as the focus of the study. As females are much more likely to experience sexism in MMOs, they might feel more compelled to answer the questions in the survey and thus skew the general statistical findings of gender distribution in *World of Warcraft*. With that being said, the statistics presented here are provided as a complement to the open-ended questions in order for the reader to gain an overall sense of the demographics and general perceptions *within* the sample. The statistics are not meant for generalizing to the whole population of *World of Warcraft* users but instead as a snapshot of what sexism might look like in *World of Warcraft*.

### Instruments and measures

A mixed methods approach with two forms of measures was used to gather data for this research. Within the survey there were both multiple-choice questions intended for statistical information as well as open-ended questions intended for the qualitative facet of this research (Creswell, 2009). Multiple choice questions included topics pertaining to gender, personal experience of sexism, witnessing another user experience sexism and if sexism in *World of Warcraft* is a problem. Open-ended questions asked for users to further describe their experiences and opinions of sexism in *World of Warcraft*. Question content included describing incidents of sexism whether against themselves or against other players, the extent to which they may or may not believe females are treated differently in the *World of Warcraft* community, general feelings about sexism in the game, if users take any actions when they see sexism happening and what users feel can be done to help the gaming culture of *World of Warcraft* to be more inclusive for women.

### Procedures

The data was gathered over a period of 1 month. This allowed participants plenty of time to take the survey. In order to analyze the findings, content analysis, coding, and statistical analyses were used. Common themes are presented in the results. Themes are presented based on the frequency of respondents that expressed similar views. Multiple responses are included to give a sense of what is typically expressed. The responses expressing incidents of sexism are plentiful, however, in the interest of being concise, responses are narrowed down to those that best represent the essence of the whole of the responses. The responses from participants are reproduced verbatim. A list of game definitions can be found in the Appendix.

### Ethical considerations

Because participants were self-selected, respondents were able to take part in this survey of their own volition. Ethical approval was sought from the home institution and was granted. The consent form included: contact information for both the researcher and the sponsoring institution, the purpose of the research, benefits for participation, restriction on age (18+), acknowledgment of risks to the participants, a guarantee of confidentiality, assurance that the participant can withdraw at any time and have the freedom to skip any of the survey questions. Participants provided no names or screennames, therefore their identity remained completely anonymous and unknown throughout the research process. All responses provided by the participants were only viewed by the researcher. I have been a subscriber to *World of Warcraft* for about 3 years and have thorough experience of game terminology, game mechanics, and game culture. The respondents were made aware of my subscription to *World of Warcraft*. Throughout the research process, I never directly interacted with the participants. I was able to analyze results from an insider perspective whereby sexism is reviewed in the context of the game.

## Results and discussion

### Multiple-choice survey results

In order to illustrate gender differences in opinion and experience, a basic crosstabulation was performed. Crosstabulation was chosen as it provided a descriptive look at the relationship between two variables. In running crosstabs, gender was the independent variable. The three questions regarding personal experience, experience of others, and the opinion if sexism is a problem or not were held as the dependent variables. When reporting on personal experience of sexism in the game, 11.6% (*n* = 19) of males reported they have experienced sexism while females within the sample were much more likely to have experienced sexism with 63.6% (*n* = 75) of females reporting yes (*Cramer's V* = 0.544, *p* < 0.001). When the respondents were asked if they had seen another player experience sexism within the game, 56.5% (*n* = 91) of males answered yes and 75.2% (*n* = 88) of females reported yes (*Cramer's V* = 0.193, *p* < 0.001). 27.5% (*n* = 44) of males reported sexism as a problem in the game whereas a total of 45.3% (*n* = 53) of females responded yes (*Cramer's V* = 0.184, *p* < 0.01). In the entire sample, 64.4% (*n* = 183) believed that sexism was not an issue in the game whereas 35.6% (*n* = 101) reported it as an issue.

### Open-ended survey results

While plenty of respondents expressed views, opinions, and blatant experiences of sexism, not all respondents believed sexism was an issue or experienced it in the same ways. However, the point of the research is to display and discuss the sexism that is *occurring*. Regardless of opinion, sexism is occurring in MMOs (overwhelmingly toward females) and it merits discussion (King et al., 1991; Herring, 1999; Bertozzi, 2008; Dill et al., 2008; Jenkins and Cassell, 2008; Gray, 2012; Salter and Blodgett, 2012; O'Leary, 2012; Braithwaite, 2013).

### Exclusion

Exclusion is by far the most prominently mentioned issue in the whole of the responses. Within the sample, female players were often seen as a detriment to game play and removed or isolated from the gameplay by other players. Removal and exclusion were enacted with several justifications such as the belief that female players cause drama as well as their perceived inability to play the game well. Many respondents reported experiences whereby an identification of a female game leader resulted in harassment, isolation and exclusion:

I have been in raid and when the rest of the raiders found out that raid leader was female the majority of them left group. Another instance was when our group was having trouble downing a particular raid boss and a female raider had a suggestion (who had downed that particular boss). I feel the suggestion was completely dismissed because of her gender.

Often, negative character traits such as simply being female and the stereotype of females causing drama were cited as reasons why a female player might be excluded:

Not being comfortable to raid lead at times because I have noticed that some males just won't listen due to a female playing a game (that seems male dominant). I have even been told or have had other female friends been told that “females can't play this game.” Have seen guild recruitment state that they do not recruit female players due to drama that they may cause.

Once I healed a dungeon and the group said what a great job I was doing. It later was discovered I was female and they were astonished that I “could actually play well with those tits in your way” (their words).

Respondents also discussed the pervasive perception that female players do not have leadership skills and therefore should not be trusted to lead group activities in the game:

This incident occurred a few years back in an ICC pug/guild run. The voice chat client Ventrilo was required for the run. No one in the guild had any raid lead experience, so they asked the p.u.g. players if one of us would lead. I stepped up because I had a thorough knowledge of the fights from clearing ICC on my main. When I first started instructing our group on the fights they were surprised by my voice. (“Wow a girl!”) but the first few bosses went smoothly. as the night went on the rest of the group started making comments about my gender. like “(Tank) don't let her tell you what to do, are you ^*****^-whipped?” and other comments about my leadership skills based purely on my gender. When we got to the princes fight, some jerk thought it would be funny to start moaning suggestively every time I talked. then all the other guys joined in. After this I couldn't stand it anymore and I just turned the computer off. I don't speak in Ventrilo anymore.

There is a stigma that women can't perform class roles as well as men. I encountered resistance constantly as a tank during ICC pug raids. I was working on my Shadowmourne quest line on my Deathknight. I had a core group of a dozen people who would raid with me weekly and I would have to recruit the other half through public channels. Once the group was assembled and summoned to the raid, I would explain over vent and on the in-game chat loot rules and scheduling (how fast we would be moving and when breaks would occur). Invariably someone would take exception to having a woman leading or tanking. I would lose between one and three people for this weekly.

Comments suggesting the normative maleness of the *World of Warcraft* environment support the previously discussed idea of hypermasculinity. Often identity traits other than masculinity are used as insults and are made to feel unwelcome:

It feels like men think they own the game and the gaming experience. A lot of them don't want women around. Trade chat is constantly full of people slamming women and alienating women from playing the game. Who wants to play with a bunch of people like that? Jokes typically have gay men, and straight women as their punchlines. Insults seem to work the same way. You never hear someone say, “Wow, you must be a man LOL” when insulting someone, because in this environment that isn't an insult.

The belief, as discussed by previous research that females do not play games such as MMOs may perpetuate this idea that females are particularly sub-par at playing these games. The assumption may be that the female players may have less experience with the game and therefore will not play the game well. Interestingly, the respondents were rarely given the opportunity to prove if they were good players; it was automatically assumed that they were bad. Typically, in U.S. society, males are expected to be competitive in nature while females feel this pressure to a lesser degree (Messner, 2002). With the idea that females are “inherently” less competitive, it might be assumed that they will not be as exemplary at competitive or challenging activities. In addition, women are both a smaller portion of the player base and also more likely to hide their gender. It would not be far-fetched to say that males might be playing with a particularly skilled female player, but her gender is often hidden. Because females are a minority and are expected to be worse players, their skills are scrutinized to a greater degree than their male counterparts. So in essence, if female players do not perform perfectly, they are judged more harshly. Females often expressed in their responses that even if their skill was proven, their success was attributed to some outside source, not the players themselves. As one respondent stated, “Back in Cata when I was in full DS heroic epics, people I would encounter outside my guild would assume I was carried no matter how hard I stomped them on the meters.” The stereotype of women being bad players could be, in itself, a strong force for exclusion. Additionally, several female respondents expressed isolating incidents in which they were made to feel unwelcome in the *World of Warcraft* community:

[…] and then there are responses that I find particularly disturbing, such as “there are no women on the internet,” which, harmless as it may seem, has a rather isolating effect. Its purpose is to say, “you don't belong here.”

A friend was told she should uninstall due to being a female and would never know how to play a game because she didn't have a “dick.”

I feel like women aren't seen as “real” gamers. There has to be some external reason they're playing a game, whether it's to express themselves as different from other women or to impress a boyfriend, they can't enjoy the game for what it is.

As discussed in the literature review, the gaming community is often seen to be a masculinized environment. Having more females in the game challenges this perception and expressions of femininity are often met with hostility and dismissal. Normative maleness is challenged when the player base sees more female gamers which some males will find threatening. The resulting hostile environment is a reason why many female players might feel discouraged from playing the game or choose to leave guilds/raids/servers, etc.

### Gendered flaming/griefing

As explored in the literature pertaining to cyberbullying and based on the responses collected, it can be theorized that cyberbullying in MMOs often presents itself in the form of gendered flaming/griefing. We can recall that griefing refers to the use of the gaming environment/mechanics to cause distress and, as I will argue here, it can also be used to exclude players. Flaming differs in that it is often insults and derogatory language that are, in this case, aimed at the female gender. Female players often express instances of flaming/griefing because of their gender, but do not specifically identify it as flaming or griefing:

There's too many incidents to put out here. Being told I must suck because I'm a girl, being told that I should be on my knees giving oral sex to my man because that's my place, being told that I should be in the kitchen making food for my man and not on WoW […]

I was forced to leave a guild and transfer off of a server due to immature sexism from a guild member. I had someone constantly tell me I smelled like fish, many times over the months. Whispers, raid chat, Ventrilo. My guild leader refused to do anything, so I left.

[…] The next guild I joined I was harassed by one particular player who would ask me sexual questions, I would refuse to answer and he screenshotted it and put it on the guild forum with ‘translations’ beneath about what I really meant. I asked the guild master to put a stop to it but he refused saying basically that boys would be boys and if I wanted to be treated like an equal I ought to have pretended to be male. Since then I have been much more careful about what guilds I join and in several have not revealed my gender. When they did not know I was a woman they would talk about women in horrible, degrading terms and make fairly constant rape jokes which caused me to leave another guild. Since then I have server hopped got into a lovely guild which was much nicer and not abusive. I also know a woman who tried voice chat when it first came out and got harassed over voice chat and then in whispers by people saying ‘suck my dick’ until she had to report it to the GMs who I think gave the person a ban.

From previous research we know that flaming occurs in voice chat as well as text chat. In all of these examples, derogatory language is directed at the female players. Leaving guilds, raids, servers, etc., was often used as a tactic to avoid the flaming or griefing. Griefing, in contrast to these instances of flaming, often presents itself in the form of exclusion in this context. The previous theme of exclusion highlights the power of kicking or barring women from raids, guilds, etc. as a sexist act of griefing. This prevents females from accessing parts of the game that might otherwise be easily accessible for male players. The game environment/mechanics, in these cases, are being used in a way to cause distress (Warner and Raiter, 2005; Coyne et al., 2009). Often griefing and flaming are used together to cause distress for female players. If we are to assume based on current research that flaming and griefing are a form of cyberbullying in the online gaming world, we could then hypothesize that sexist forms of flaming and griefing are forms of cyberbullying as well.

### Identity concealment

Many of the respondents, specifically female respondents, expressed a need for concealing their identity for the purpose of protection from harassment:

A friend of mine, a girl, was new to the game and decided to play with me, also a girl. We made female blood elves together and played with no incident for around half an hour. We went back to the city for something, and then had a group of 4 guys following us around and not letting us quest in peace. After a lot of hounding and interfering with our quest mobs, they convinced her to go back to the city with them and offered to pay her 100 g to take off her character's clothes and dance in one of the inns. I suggested that we make male toons and she agreed. After making male characters, we encountered no more problems. What a great first time impression of the game, eh?

There are many females I know who will not speak in vent, due to being harassed in game because they are a woman.

I choose to allow other gamers to assume I am male. I will lie about not having a microphone in PUG raids to avoid attention.

My wife also plays, and feels uncomfortable portraying herself as a woman because of the level of sexism.

Often harassment results in female players choosing to hide their identities. Bringing any sort of attention to the fact that they are female can often result in unwanted attention with negative outcomes. It is understandable then that female players do not feel comfortable sharing something so integral to their identity (gender). Male players can freely express their identity because sexism is not a huge threat to the male player along with the assumption that most players in *World of Warcraft* are males. Other theories suggest that female players might feel as though revealing their identity will result in decreased expectations or that fellow gamers will stop playing with them (Yee, 2008; Williams et al., 2009a,b; Eklund, 2011). As discussed in the previous section, decreased expectations and exclusion are a reality. A few respondents expressed that identity concealment should be a solution to issues of sexism and harassment:

If being singled out for being a woman is such an issue to the player, the option to play as a male character is available to them, or better yet don't reveal your true gender to people you don't trust.

If they don't want the attention then they would need to also not make a big deal that they are a female.

The inherent issue with solutions like this is the fact that *World of Warcraft* is a social game and players often use programs that use voice chat. Often, if a group activity is to go smoothly, it is important for players to communicate in voice chat. In order to avoid revealing their “true gender,” voice chat is generally not an option. Herein lies an example of the privilege male players enjoy in MMOs; male players generally will not expect harassment due to their gender. For females, this may make it difficult to find and build community when you can experience harassment at the sound of your own voice or discussing anything that might reveal your female gender.

### Gendered exploitation/appeasement

A comment in the survey aptly summed up the particular issue of gendered exploitation/appeasement, “you're either incompetent because you're a girl or treated with undue respect/deference.” Often respondents had discussed the fact that male players will give female players favors such as gold, mounts (collectible objects that provide transportation), pets (cosmetic pets that follow the player around), etc.:

I have a lot of females as friends, and many of the males tend to give gifts to women in order to try and gain favor.

At other times, we receive the OTHER kind of sexism—male players assuming that if they give us stuff, we'll be their girlfriends or send them naked pictures.

Other times they are worshiped, e.g., free items, dudes groveling up to them. Sometimes they are treated like they are precious and delicate (e.g., white knighting).

Some people believe that just because we're female that we can trick males into giving us items, gold or other stuff for free when that is not the case.

Several respondents complained that some females will exploit male players in an attempt to receive in-game favors. This may be the case; however, within the sample none of the female respondents indicated they had attempted to receive favors from males so we cannot definitively know for sure. On the same topic, both male and female respondents noticed that male players often felt they needed to “help” female characters/players:

However, some male players treat female players like they cannot function on their own. Some, they give the females gear and get over protective.

Some respondents expressed the belief that males would help and provide gifts in order to gain something from the female character such as cyber-sex. The phenomenon of male players providing special attention, help, or gifts has been documented before with the theory of heterosexual desire being a motivating factor (Eklund, 2011). Additionally, the perception that female players need help from male players (often called white-knighting) might go back to the stereotype that female players are bad at the game and therefore need more help which can be attributed to Gender Schema Theory and Ambivalent Sexism Theory. The idea being that we hold deeply ingrained gender stereotypes (Gender Schema Theory) and assume that female players are subordinate and therefore may need more help within the game (Ambivalent Sexism Theory) (Dill et al., 2008; Downs and Smith, 2009).

### Female character portrayal

A large number of respondents expressed distaste for how female characters are portrayed in the game. For example, female characters are by far more likely to be scantily clad (this is especially true of armor sets in the earlier years of the game):

Simply the way females are made to look “sexy” in armor is sexism. Something that's long pants on males is a thong on females for instance.

I actually commented earlier on a forum thread about the sexiness of the female characters. The shape of their bodies is laughable and ridiculous. The male bodies aren't exactly realistic either but at least they don't look like pornstars. I'm not saying the girls should be flat as a board, I'm just saying they should tone it down a bit… even just a little boob reduction would be better.

While female clothing and body proportions have gotten better as *World of Warcraft* has grown older, it is still fairly easy to find lore characters and player avatars in revealing, hypersexualized outfits. Not to mention, as one respondent stated, female character's bodies tend to be, “laughable and ridiculous.” It is obvious from the responses that many players are uncomfortable with the hypersexualization of *World of Warcraft* characters. However, hypersexualization was not the only issue cited often. Female lore characters are not only hypersexualized, but are also hard to come by; much of the lore is centered on the male storyline. When female characters are central to the lore, they tend to be dependent on male characters for their strength or they are painted as evil characters:

And compared to the male story line characters of *World of Warcraft*, there are hardly any major female characters that have much affect to the plot.

Look at the story of WoW. All the male characters are portrayed as big, self-sacrificing and heroic. The female characters are either utterly reliant on a male character or transparently shallow and evil. Players mimic this and treat women as inferior, as an addition to the general gamer misogynist environment.

As a respondent expressed, “I think the lack of strong female characters that break gender stereotypes doesn't help much either.” Many survey respondents called for “stronger female story lines and character development.” Of course, there are exceptions; the gaming industry and Blizzard are producing stronger, multidimensional female characters more often. However, because gaming companies typically cater to male players; it is no surprise that most storylines are centered on male characters.

### A more inclusive community

When asked for suggestions as to how *World of Warcraft* might be made into a more gender inclusive game, similar suggestions emerged; the first being a rethinking of how female avatars and characters are portrayed in the game:

This will sound ridiculous. It seems to me that player characters and armor items they equip, the first-glance visuals contained in the game, are very sexist. Most females are lithe and busty, and most males are muscular and brawny. It seems the exception would be Blood Elf males, and quite often I hear that Belf males ‘look’ gay. What would happen if this were to change? I have seen other games that allow customization of avatars, such as in Star Wars: The Old Republic. As to the gear, it seems that males look heavily armored, while females are scantily clad. I think it would be hilarious if sought after tanking gear would make a male avatar look like a stripper for once.

This comment points out a particular remedy for game avatars to reach beyond the trend of hypersexualization. In fact, many respondents called for the option of more customization of avatars. *Star Wars: The Old Republic*^4^, another MMO, allows the player to choose from many varying body types which moves away from the typical “lithe and busty” female avatar. This is not to say that the “lithe and busty” body type would not be an option, but instead be one representation of the female body among more realistic representations. Another solution cited by respondents is to include more dynamic female lore characters:

More female characters that are strong. Cataclysm managed to take Tyrande and marginalize her (not to mention make her simpering twit that acquiesces to orders from the men), and turned Sylvanas into a murderer with no conscience. These are not strong females.

Female lore characters that fulfill more than the stereotypical roles could be a step toward engaging more female players in the lore of *World of Warcraft*. Plenty of males in MMOs are presented as strong, dynamic characters. Female characters like this are lacking. If we are to accept the research that suggest men and women (and boys and girls) learn gendered behavior from many sources including the media, we can think about how players might learn from more dynamic and strong female characters. In the same line of thinking, players suggest more visible female game developers or community managers from Blizzard:

[…] I also think it would help to have some Developers or Community Managers identify themselves as Females to make the Women in WoW feel more inclusive.

Seeing more visible female game designers, community managers, etc. might help to address the small proportion of female MMO players but also the lack of female representation as game designers. Seeing more women involved in game design and play could allow a bridge for females to challenge gender stereotypes and cross into a typically “masculine” environment. The most prominently addressed solution for making the community more inclusive came down to placing a large amount of responsibility on Blizzard and the players themselves:

On Blizzard's end, they could take harassment more seriously. Make a zero tolerance policy (so long as there's proof in game) and ban people. The ‘penalty volcano’ takes way too long to do anything. On the end of players… they can realize that men and women are, on a basic level, the same and no one is inferior to anyone. But I don't think that'll happen any time soon.

Blizzard could implement a system for rating players attitudes and behaviors in public qued content, to help identify and deal with problem players, similar to those starting to be employed by games like DOTA2. It seems like a social issue among gamers, not something caused by Blizzard's game design, however.

Blizzard needs to take sexism seriously. Other players need to call people out on their sexist comments. Change has to come from the inside.

One of the main suggestions made to combat these issues is for Blizzard to take harassment and sexism more seriously. It is hard to say how seriously Blizzard punishes offenders for cyber-bullying, harassment, sexism, etc. because they do not release that information. Generally, if the perpetrator abuses policies more than once, the punishment becomes more severe and might eventually result in a strong sanction, such as a significant ban from the game. Often respondents called for a zero-tolerance policy whereby any sexist remarks or actions do not result in a warning but rather a more serious consequence such as a significant ban. The theory of online disinhibition points to the minimization of status and authority in this online world. When appearances of authority are minimized, consequences for offensive and hurtful behavior are not perceived to be a threat. Therefore, players are more willing to misbehave (Suler, 2004). Respondents also believed what has been discussed before; that it is not the game content itself that makes inclusivity challenging, but rather the game culture or the actions and behaviors of other players (Yee, 2008). As some respondents pointed out, anonymity on the internet allows players to act out:

Honestly, I think the anonymity of the internet is the larger issue. People express what they think without regard toward others, and that sort of attitude “spills over” into *World of Warcraft*. […]

According to Suler (2004), we might attribute these actions to what is called the online disinhibition effect. In this context, we would name this as toxic disinhibition. Players have the opportunity to separate their actions from their real world lifestyle and identity and feel less inhibited to act out. This is what is known as dissociative anonymity. Suler puts it best, “In a process of dissociation, they don't have to own their behavior by acknowledging it within the full context of an integrated online/offline identity” (Suler, 2004, p. 322). This is key, players do not have to own their behaviors; they are rarely responsible for consequences that result from their sexist behaviors.

## Conclusion

Although anonymous surveys can be a strong tactic in research such as this, respondents had the opportunity to lie or intentionally skew the findings. Sexism in gaming is an extremely contentious topic therefore players might want to make a point that sexism is or is not an issue and respond in a way that supports their point of view even if their responses are not factual. Some respondents expressed concerns that the research was predominately searching for sexism in the game itself, however, the goal was to focus on the sexist attitudes of the player base as well as possible sexism within the game design. Wording in the survey questions might have misled players to only discuss issues within the game design itself. One point that must be addressed and has been touched on in the research, is the concern expressed by players that the research might be biased because it is focused on females experiencing sexism rather than males. The goal of the research is to study where the majority of the issue lies. History, previous research, and recent research have all shown that sexism is experienced much more frequently by females than males. However, it is true that males will experience sexism to a lesser degree (and often experience racism, homophobia, etc.). Future research might look at what sort of sexism these males are experiencing, who is perpetrating it and why it is happening. The topic of gender in technology is full of interesting research opportunities. A study might examine more in depth why many respondents do not deem sexism to be a problem in MMOs even though it is clearly experienced by a large amount of female gamers. Research might also focus on the treatment of other minority groups in online games such as race, sexual orientation, etc. There is also plenty of opportunity to study how cyberbullying presents itself in online gaming and how gender interplays with the topic. These are only a few suggestions as there are many avenues for research on this topic.

The aim of this exploratory study is to provide attitudes and experiences, perceptions and opinions about sexism in the online game, *World of Warcraft*. However, it is beneficial to discuss and theorize why certain themes and statistics arose from the data. It should be stated that while the statistics show that 64.4% of the sample chose to report that sexism is not an issue in the game, the responses of those that did report and describe it as an issue is significant enough that it can be deduced that many player's lives are being affected negatively by sexism. The open-ended questions revealed reoccurring themes surrounding sexism and harassment, such as exclusion, identity concealment, gendered exploitation, cyberbullying in the forms of flaming and griefing, female character portrayal and common opinions on how to make *World of Warcraft* (whether it be how the game is designed or the player culture) more inclusive. The research shows that while not all females who play MMOs will experience sexism, many do. Whether respondents view sexism as an issue or not, it is still happening and should be discussed.

One takeaway other researchers and myself have documented is that sexism in gaming can be a strong deterrent for females in an already male dominated medium. In order to make games more inclusive and enticing for women, sexism should be addressed, discussed, and therefore, steps should be taken to improve it. As highlighted before, many people argue that because this sexism is occurring online, it is not a real problem. Cyber-bullying has recently emerged as an issue that people are taking steps to solve. Sexism in gaming is debatably a form of cyber-bullying with real-life consequences. Moreover, online worlds reflect *real* societal processes, norms, prejudices, etc. If we cannot address sexism online, how can we hope to tackle it in the “real” world?

Gamers often point out a debate that asks how the harassment of men online is different from the harassment of women. We must first acknowledge that men are rarely harassed due to their gender. This is not to say that general harassment directed at men is not psychologically harmful; that particular subject can be explored in future research. Instead, this is to say that women's gender identities are often specifically targeted. The literature has made it clear that the targeting of gender and more specifically sexism does have a psychological effect on female players. Indeed, the research findings presented here reveal that female players can be affected psychologically, ranging from feelings of exclusion to feelings of distaste for the players perpetrating sexist attitudes. The topic of psychological distress of female gamers who experience sexism online could also add to the literature surrounding the treatment of females in the online world. One aspect of this debate is without doubt, the findings overwhelmingly indicate that females cannot often fully enjoy aspects of the game because of the reactions of other players to the female gender. We see that females can be barred/kicked out of guilds, harassed in voice chat, removed from raids, removed or ignored in leadership positions along with a myriad of sexist attitudes and actions that can make the game less enjoyable. Here I challenge game developers to find creative ways to lessen the amount of sexism that is allowed to flourish in online games, but perhaps more importantly, I suggest that players hold themselves responsible for not only changing their own behaviors but for also calling out the toxic behaviors of other players. Pressure from the playerbase could result in less toxic behavior. Only then can female gamers take part in the shared privilege of a positive gaming experience so that they can more freely enjoy slaying digital foes.

### Conflict of interest statement

The author declares that the research was conducted in the absence of any commercial or financial relationships that could be construed as a potential conflict of interest.

## Footnotes

^1^http://www.businesswire.com/news/home/20130725006767/en/Activision-Blizzard-Announces-Transformative-Purchase-Shares-Vivendi

^2^Blizzard Entertainment. (2004-present). The World of Warcraft. Irvine, CA.

^3^http://fatuglyorslutty.com/

^4^LucasArts. (2012). Star Wars: The Old Republic. CA: San Francisco.

## Appendix

### Definitions of commonly used words in *world of warcraft*

Blood Elves—A playable race in *World of Warcraft.*

Carried—Players who are not able to complete objectives themselves and need help from others.

DOTA 2—A separate game from *World of Warcraft* known as Defense of the Ancients, a multiplayer online battle arena.

DS—Dragon Soul, a raid/instance (See: Raid).

Dungeon—A five player instance/area where players cooperate to defeat various computer controlled encounters.

Epics—Gear which is more powerful and harder to get than normal gear.

Gear/Armor—Equipment that characters use to become more powerful. Better gear is one of the game's main rewards.

Guild/Guildies—A player formed and governed community within the game. Guildies are guild mates.

Heroic—Instances which are harder than normal raids and dungeons and provide better rewards.

ICC—Icecrown Citadel, a raid/instance (See: Raid).

Loot—Rewards that can be obtained by players such as armor/gear.

Lore—A continuing story being played out in the *World of Warcraft.*

Meters—A tool to measure various performance markers such as damage done.

MMORPG—Massively Multiplayer Online Roleplaying Game.

Mobs—A computer controlled entity (such as monsters) within the game, usually hostile.

PUG—Pick up group, finding players (usually through community chat channels) to group up and complete objectives.

Quest—Objectives given within the game that often come with a reward (e.g., gear, experience, etc.)

Raid—An area/instance meant for larger groups of players to cooperate to defeat various computer controlled encounters. Similar to dungeons but are more difficult and have better rewards.

Sylvanas—A primary lore character in *World of Warcraft.*

Tank—A player who takes on a leadership role, takes the brunt of the damage in encounters and prevents other players from being attacked.

Trade Chat—A general chat channel within the game.

Toons—Shorthand for characters.

Tyrande—A primary lore character in *World of Warcraft.*

Vent/Ventrilo—A third-party program that allows voice communication between players.

### Survey questions

#### Multiple-choice questions

What is your gender?

➢Male
➢Female
➢Other (I do not identify as either male or female.)

What is your age group?

➢18–20
➢21–25
➢26–30
➢30–50+

Have you personally experienced an act of sexism in *World of Warcraft* as a cause of your gender?

➢Yes
➢No

Have you seen someone else experience sexism in *World of Warcraft*?

➢Yes
➢No

Do you think sexism in *World of Warcraft* is a problem?

➢Yes
➢No

#### Open-ended questions

➢Have you or anyone you know ever experienced instances of sexism in game, over voice chat, or on the forums? If comfortable, please describe the incident.
➢To what extent do you feel that female players are treated differently in the game than male players? Why do you feel this way (personal experience, experience of a friend, observation of others, etc.)?
➢How do you feel about sexism in the game, over voice chat, or on the forums? Has it affected you personally?
➢What do you think could be done in order to make *World of Warcraft* more inclusive for women?
➢When you see sexism in game, over voice chat, or on the forums, do you feel as though you should do something about it? If so, what do you do?
➢If you have any more comments pertaining to this subject or other questions please feel free to post them.

## References

[B2] Anderson C. A. (2004). An update on the effects of playing violent video games. J. Adolesc. 27, 113–122 10.1016/j.adolescence.2003.10.009 15013264

[B3] Bandura A. (1965). Influence of model's reinforcement contingencies on the acquisition of imitative responses. J. Pers. Soc. Psychol. 1, 589–595 10.1037/h0022070 14300234

[B4] Beasley B. Standley T. C. (2002). Shirts vs. skins: clothing as an indicator of gender role stereotyping in video games. Mass Commun. Soc. 5, 279–293 10.1207/S15327825MCS0503_3

[B5] Bertozzi E. (2008). ‘You play like a girl!’: cross-gender competition and the uneven playing field. Convergence Int. J. Res. New Media Technol. 14, 473–487 10.1177/1354856508094667

[B7] Braithwaite A. (2013). ‘Seriously, get out’: feminists on the forums and the war(craft) on women. New Media Soc. 15, 1–16 10.1177/1461444813489503

[B8] Bryce J. Rutter J. (2003). Gender dynamics and the social and spatial organization of computer gaming. Leisure Stud. 22, 1–15 10.1080/02614360306571

[B9] Connell R. W. (1995). Masculinities. Berkeley, CA: University of California Press

[B10] Coyne I. Chesney T. Logan B. Madden N. (2009). Griefing in a virtual community: an exploratory survey of second life residents. J. Psychol. 217, 214–221 10.1027/0044-3409.217.4.214

[B11] Creswell J. W. (2009). Research Design: Qualitative, Quantitative, and Mixed Methods Approaches. Thousand Oaks, CA: Sage Publications, Inc

[B12] Dietz T. L. (1998). An examination of violence and gender role portrayals in video games: implications for gender socialization and aggressive behavior. Sex Roles 38, 425–442 10.1023/A:1018709905920

[B13] Dill K. E. Brown B. P. Collins M. A. (2008). Effects of exposure to sex-stereotyped video game characters on tolerance of sexual harassment. J. Exp. Soc. Psychol. 44, 1402–1408 10.1016/j.jesp.2008.06.002

[B14] Dill K. E. Thill K. P. (2007). Video game characters and the socialization of gender roles: young people's perceptions mirror sexist media depictions. Sex Roles 57, 851–864 10.1007/s11199-007-9278-1

[B15] Dohnt H. Tiggemann M. (2006). The contribution of peer and media influences to the development of body satisfaction and self-esteem in young girls: a prospective study. Dev. Psychol. 42, 929–936 10.1037/0012-1649.42.5.929 16953697

[B16] Downs E. Smith S. L. (2009). Keeping abreast of hypersexuality: a video game character content analysis. Sex Roles 62, 721–733 10.1007/s11199-009-9637-1

[B17] Durkin L. (1985). Television and sex role acquisition. Br. J. Soc. Psychol. 24, 101–113 10.1111/j.2044-8309.1985.tb00669.x 3893609

[B18] Eklund L. (2011). Doing gender in cyberspace: the performance of gender by female *World of Warcraft* players. Convergence Int. J. Res. New Media 17, 323–342 10.1177/1354856511406472

[B19] ESA, (2010). Sales, Demographics, and Usage Data: Essential Facts About the Computer and Video Game Industry. Washington, DC: Entertainment Software Association Available online at: http://www.theesa.com/facts/pdfs/ESA_Essential_Facts_2010.PDF

[B20] ESA, (2013). Sales, Demographics, and Usage Data: Essential Facts About the Computer and Video Game Industry. Washington, DC: Entertainment Software Association Available online at: http://www.theesa.com/facts/pdfs/ESA_EF_2013.pdf

[B22] Graham R. (2012). The Attack on Anita Aarkeesian: From Media Analysis to Anti-Feminism and Online Harassment. Available online at: http://thesocietypages.org/sociologylens/2012/07/02/the-attack-on-anita-sarkeesian-from-media-analysis-to-anti-feminism-and-online-harassment/

[B23] Gray K. L. (2012). Intersecting oppressions and online communities: examining the experiences of women of color in xbox live. Inform. Commun. Soc. 15, 411–428 10.1080/1369118X.2011.642401

[B24] Herring S. (1999). The rhetorical dynamics of gender harassment on-line. Inform. Soc. 15, 151–167 10.1080/019722499128466

[B25] Jenkins H. Cassell J. (2008). From quaker grrls to desperate housewives: a decade of gender and computer games, in Beyond Barbie and Mortal Combat: New Perspectives on Gender and Gaming, eds Kafai Y. B. Heeter C. Denner J. Sun J. Y. (London: MIT Press), 5–19

[B26] Kayany J. M. (1998). Contexts of uninhibited online behaviour: flaming in social newsgroups on Usenet. J. Am. Soc. Inform. Sci. 49, 1135–1141 10.1002/(SICI)1097-4571(1998)49:12<1135::AID-AS18>3.0.CO;2-W

[B27] Kendall L. (2000). “Oh no! i'm a nerd!”: hegemonic masculinity on an online forum. Gender Soc. 14, 256–274 10.1177/089124300014002003

[B28] Kennedy H. W. (2002). Lara croft: feminist icon or cyberbimbo? On the limits of textual analysis. Game Stud. 2 Available online at: http://www.gamestudies.org/0202/kennedy/

[B29] King W. Miles E. Kniska J. (1991). Boys will be boys (and girls will be girls): the attribution of gender role stereotypes in a gaming situation. Sex Roles 25, 607–623 10.1007/BF00289567

[B30] Kuznekoff J. H. Rose L. M. (2012). Communication in multiplayer gaming: examining player responses to gender cues. New Media Soc. 15, 541–556 10.1177/1461444812458271

[B32] Malpas J. (2009). On the non-autonomy of the virtual. Convergence 15, 135–139 10.1177/1354856508101579

[B33] Markey P. M. Markey C. N. (2010). Vulnerability to violent video games: a review and integration of personality research. Rev. Gene. Psychol. 14, 82–91 10.1037/a0019000

[B34] Messner M. (2002). Taking the FIeld: Women, Men, and Sports. Minneapolis, MN: University of Minnesota Press

[B35] O'Leary A. (2012). In virtual Play, Sex Harassment is all too Real. The New York Times. Available online at: http://www.nytimes.com/2012/08/02/us/sexual-harassment-in-online-gaming-stirs-anger.html?_r=0

[B36] Parrott D. J. Zeichner A. (2008). Determinants of anger and physical aggression based on sexual orientation: an experimental examination of hypermasculinity and exposure to male gender role violations. Arch. Sex. Behav. 37, 891–901 10.1007/s10508-007-9194-z 17680354

[B12a] Renzetti C. M. Curran D. J. (2003). Women, men and society 5th ed. Boston, MA: Allyn & Bacon

[B37] Royse P. Lee J. Undrahbuyan B. Hopson M. Consalvo M. (2007). Women and games: technologies of the gendered self. New Media Soc. 9, 555–576 10.1177/1461444807080322

[B38] Salter A. Blodgett B. (2012). Hypermasculinity and dickwolves: the contentious role of women in the new gaming public. J. Broadcast. Electron. Media 56, 401–416 10.1080/08838151.2012.705199

[B39] Suler J. (2004). The online disinhibition effect. CyberPsychol. Behav. 7, 321–326 10.1089/1094931041291295 15257832

[B40] Taylor N. Jenson J. de Castell S. (2009). Cheerleaders/booth babes/halo hoes: pro-gaming, gender and jobs for the boys. Digital Creativity 20, 239–252 10.1080/14626260903290323

[B41] Taylor T. L. (2003). Multiple pleasures: women and online gaming. Convergence 9, 21–46 10.1177/135485650300900103

[B42] Taylor T. L. (2006). Play between Worlds: Exploring Online Game Culture. Cambridge, MA: MIT Press

[B43] Tiggemann M. McGill B. (2004). The role of social comparison in the effect of magazine advertisements on women's mood and body dissatisfaction. J. Soc. Clin. Psychol. 23, 23–44 10.1521/jscp.23.1.23.26991

[B44] Warner D. E. Raiter M. (2005). Social context in massively-multiplayer online games (MMOGs): ethical questions in shared space. Int. Rev. Inform. Ethics 4, 46–52 Available online at: http://fiz1.fh-potsdam.de/volltext/ijie/06142.pdf

[B45] Williams D. Consalvo M. Caplan S. Yee N. (2009a). Looking for gender: gender roles and behaviors among online gamers. J. Commun. 59, 700–725 10.1111/j.1460-2466.2009.01453.x

[B46] Williams D. Martins N. Consalvo M. Ivory J. (2009b). The virtual census: representations of gender, race and age in video games. New Media Soc. 11, 815–834 10.1177/1461444809105354

[B47] Yee N. (2008). Maps of digital desires: exploring the topography of gender and play in online games, in Beyond Barbie and Mortal Combat: New Perspectives on Gender and Gaming, eds Kafai Y. B. Heeter C. Denner J. Sun J. Y. (London: MIT Press), 82–95

